# Bilateral primary nonrefluxing unobstructed megaureter in an adult: a case report and review of the literature

**DOI:** 10.1186/s13256-025-05603-6

**Published:** 2025-10-29

**Authors:** Oadi N. Shrateh, Sarah Nafea, Fahad Khan, Fawad Ali, Muhammad Faheem, Naeem Sheikh

**Affiliations:** 1 Faculty of Medicine, Al-Quds University, Jerusalem, Palestine; 2 Department of Acute Medicine, Basildon University Hospital, Mid and South Essex NHS Foundation Trust, Basildon, UK; 3 Department of Urology, Basildon University Hospital, Mid and South Essex NHS Foundation Trust, Basildon, UK

**Keywords:** Primary megaureter, Bilateral, Nonobstructive, Nonrefluxing

## Abstract

**Background:**

Bilateral primary nonrefluxing unobstructed megaureter is a rare condition in adults, often identified during evaluation for nonspecific symptoms. Primary nonrefluxing unobstructed megaureter is more commonly diagnosed in pediatric populations, typically through antenatal imaging.

**Case presentation:**

A 43-year-old South Asian male presented with persistent dull left loin pain lasting 2 weeks. His medical history consisted solely of recently diagnosed type 2 diabetes mellitus, well-controlled with metformin. Imaging studies revealed bilateral distal ureteral dilatation accompanied by hydronephrosis in both kidneys. A computed tomography urogram demonstrated dilated extrarenal pelvis and ureters, with no evidence of obstruction or secondary causes. A mercaptoacetyltriglycine (MAG)-3 renal scan confirmed normal renogram curves, leading to the diagnosis of bilateral primary nonrefluxing unobstructed megaureter. The patient was managed conservatively with regular follow-up, given the absence of significant complications.

**Conclusion:**

Primary nonrefluxing unobstructed megaureter, while often resolving spontaneously in pediatric cases, requires careful diagnostic evaluation in adults to exclude secondary causes. Conservative management is appropriate in asymptomatic patients or those without significant functional impairment. This case emphasizes the value of comprehensive imaging, including ultrasound, computed tomography and renal scintigraphy, in identifying primary nonrefluxing unobstructed megaureter and guiding management. Long-term follow-up remains essential to monitor for potential complications. Although rare, it is crucial to consider primary megaureter in adult patients presenting with renal-related issues, recognizing that, in exceptionally rare cases, it may present as bilateral, nonobstructive, and nonrefluxing.

## Background

Bilateral primary nonrefluxing unobstructed megaureter (PMU) is a rare condition in adults, typically diagnosed in pediatric populations through antenatal imaging. The term megaureter refers to a ureteral dilation of > 7 mm [1], which does not imply a specific diagnosis or etiological evaluation, as it can result from various uropathies, including vesicoureteral reflux (VUR), duplicated collecting systems, ureterocoele, ectopic ureter, or bladder outlet obstruction.

Using the international classification of Smith, megaureters are categorized as primary or secondary on the basis of the underlying cause of dilation, which may be intrinsic or associated with another urinary tract pathology [2, 3]. Primary megaureter is a common condition among congenital uropathies [4–6], characterized by an enlarged ureteral diameter that may or may not be accompanied by pelvicalyceal dilatation, resulting from an anomaly at the vesicoureteral junction [4, 5]. These megaureters can be classified as refluxing, nonrefluxing, obstructive, or nonobstructive, classifications that significantly influence clinical management strategies [3, 7].

While PMU is more frequently observed in males and predominantly affects the left ureter, its occurrence in adults remains rare. In addition, although megaureter is commonly recognized in pediatric populations, the occurrence of bilateral, primary, nonrefluxing, and nonobstructive megaureter in adults is exceedingly rare. Most cases identified in adults are unilateral or associated with secondary causes such as vesicoureteral reflux, obstruction, or infection. This case is distinctive in that it presents a bilateral form without any secondary complications, managed entirely in an outpatient setting. The case emphasizes the diagnostic challenges in adult patients where the presentation may be vague and highlights the importance of a structured and noninvasive diagnostic pathway. Our report aims to fill a gap in adult urological case literature by reinforcing best practices in diagnosis and conservative management without over-reliance on invasive procedures.

## Case presentation

A 43-year-old South Asian male presented with persistent dull left loin pain lasting for 2 weeks. His medical history consisted solely of newly diagnosed type 2 diabetes mellitus, which was well controlled with metformin. There was no relevant family history, and social history was unremarkable. The patient denied any lower urinary tract symptoms (LUTS) such as dysuria, frequency, urgency, or nocturia. He also reported no history of urinary tract infections, hematuria, fever, or flank tenderness. There were no constitutional symptoms such as weight loss, night sweats, or fatigue. He had no prior history of urological interventions or similar complaints throughout adulthood or as a child. On systemic review, there were no signs suggestive of neurogenic bladder or metabolic abnormalities. Physical examination was unremarkable; there was no costovertebral angle tenderness, palpable masses, or suprapubic distension. His vital signs were within normal limits. Laboratory investigations revealed a serum creatinine of 69 µmol/L and an estimated glomerular filtration rate (eGFR) of 108.5 mL/min/1.73 m^2^. His HbA1c was 45 mmol/mol, reflecting newly diagnosed type 2 diabetes mellitus under good control. Urinalysis showed no evidence of infection or proteinuria, with negative leukocyte esterase, nitrites, and no hematuria.

**Fig. 1:**
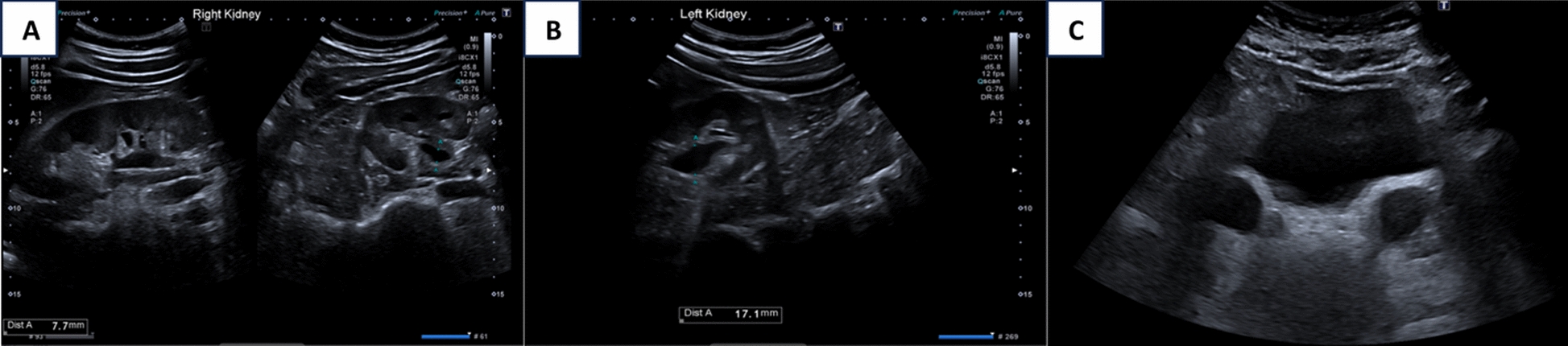
The ultrasound images of the kidneys and bladder. In **A**, the right kidney shows hydronephrosis with a renal pelvis measuring 1.5 cm in anteroposterior diameter. After emptying the bladder, the anteroposterior diameter decreased to 0.7 cm. In **B**, the left kidney shows mild hydronephrosis with a renal pelvis measuring up to 1.7 cm in anteroposterior diameter. After emptying the bladder, the anteroposterior diameter of the pelvis did not change. In **C**, the bladder is well distended and demonstrates no focal or diffuse wall thickening or trabeculations. In addition, there is bilateral distal ureteral dilatation measuring 2.5 cm on the right side and 1.8 cm on the left side, which did not resolve even after emptying the bladder

**Fig. 2:**
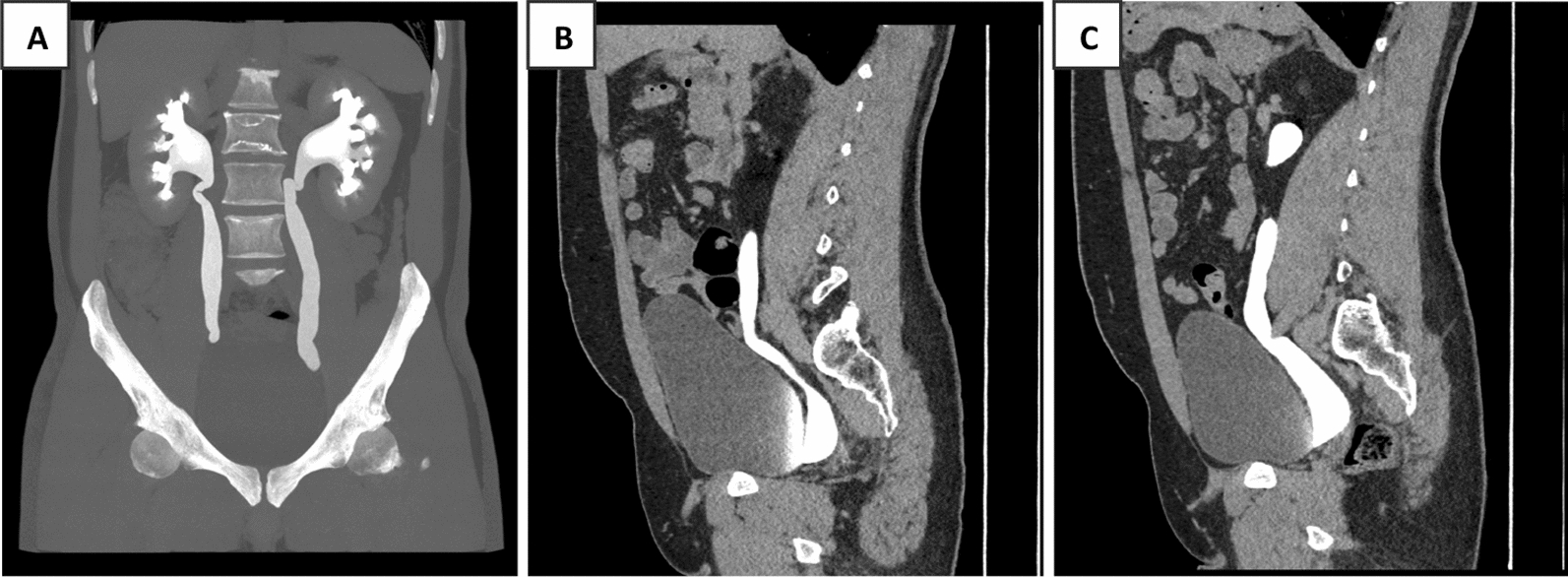
Demonstrates a contrast-enhanced computed tomography of kidneys, ureters, and bladder with anteroposterior views in (**A)**, right (**B**), and left (**C**), demonstrating a few small parenchymal cysts in the right kidney measuring up to 7 mm. No focal lesions are seen in the left kidney. The right kidney is normal in size, with no hydronephrosis and no renal calyx stones observed. The left kidney is normal in size but shows mild fullness of the collecting system without obvious renal calyx stones. There is bilaterally dilated extrarenal pelvis with dilated ureters, and the left-sided ureter is mildly prominent. The contrast is seen to flow normally within the proximal and distal hydroureters up to the urinary bladder without wall thickening, filling defects, or extrinsic compression. Impression: there is bilaterally dilated extrarenal pelvis with dilated ureters, and the left-sided ureter is mildly prominent. The contrast flows normally within the proximal and distal hydroureters bilaterally up to the urinary bladder without wall thickening, filling defects, or extrinsic compression. There is no obvious cause for the hydroureters bilaterally on this CT examination

**Fig. 3:**
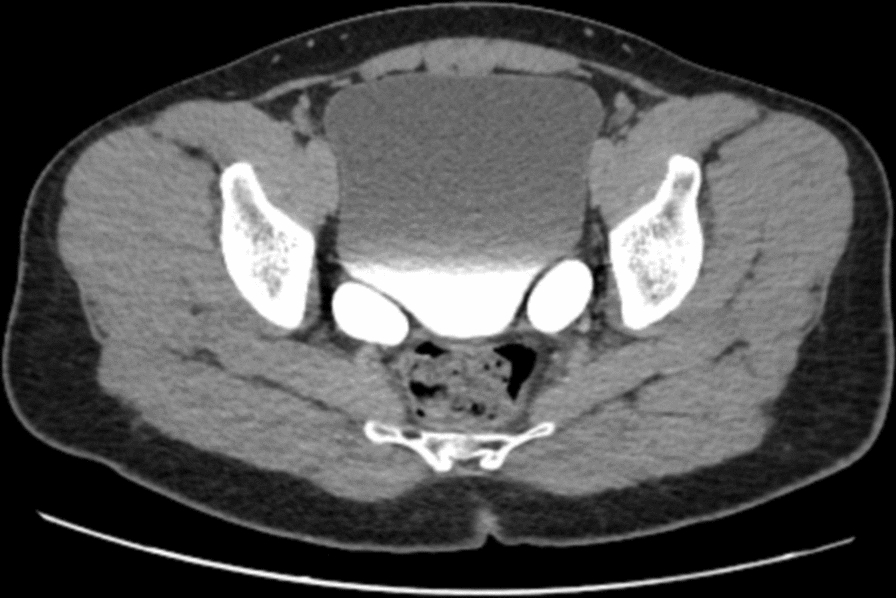
A contrast-enhanced computed tomography of kidneys, ureters, and bladder (axial section). Urinary bladder is optimally distended, thin-walled, underfilled with contrast without filling defect. Prostate gland is normal in size. Seminal vesicles are normal in appearance. No bowel obstruction or collection. No lymphadenopathy and no free fluid in abdomen and pelvis. No large mass seen in the liver, kidneys, spleen, the adrenal glands and pancreas. No destructive bony lesions seen in the visualized bony structures

Initial imaging commenced with an ultrasound of the kidneys, ureters, and bladder (US KUB). The ultrasound revealed bilateral distal ureteral dilatation, measuring 2.5 cm on the right and 1.8 cm on the left. In addition, hydronephrosis was noted in the right kidney, with a renal pelvis measuring 1.5 cm in anterior–posterior diameter, which reduced to 0.7 cm postvoid. The left kidney displayed mild hydronephrosis, with a renal pelvis measuring 1.7 cm, which remained unchanged postvoid (Fig. 1). To further investigate these findings, a CT urogram was performed. The CT urogram demonstrated bilaterally dilated extrarenal pelvis and ureters without wall thickening or filling defects (Figs. 2 and 3**).** No specific obstructive cause for the hydroureters was identified.

**Fig. 4:**
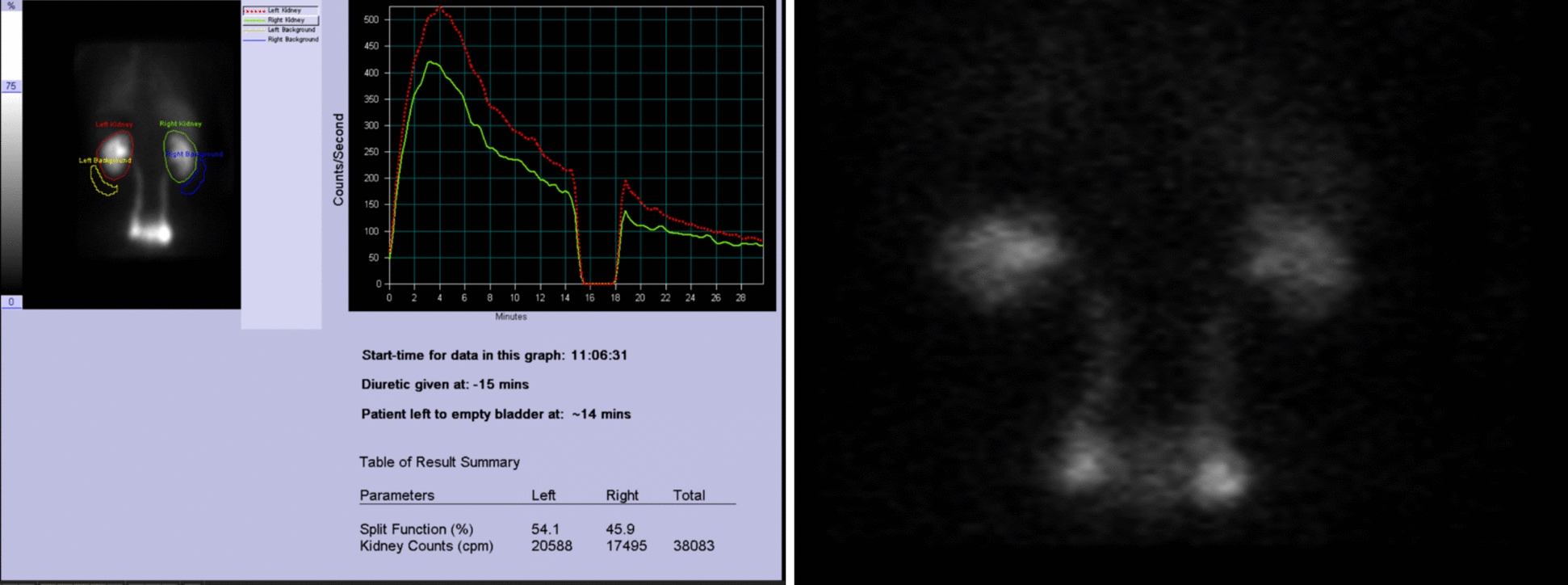
A mercaptoacetyltriglycine (MAG) 3 renogram. The renogram curves are normal in shape. Despite the bilaterally dilated ureters there is no suggestion of any obstruction. Likely bilateral megaureters but with no obstruction

To confirm the functional significance of the findings, a renal scan with a diuretic (MAG-3 renogram) was performed. The results revealed normal renogram curves with no evidence of obstruction, despite the bilateral ureteral dilation (Fig. 4). These findings confirmed the diagnosis of bilateral primary megaureter (PMU).

**Fig. 5:**
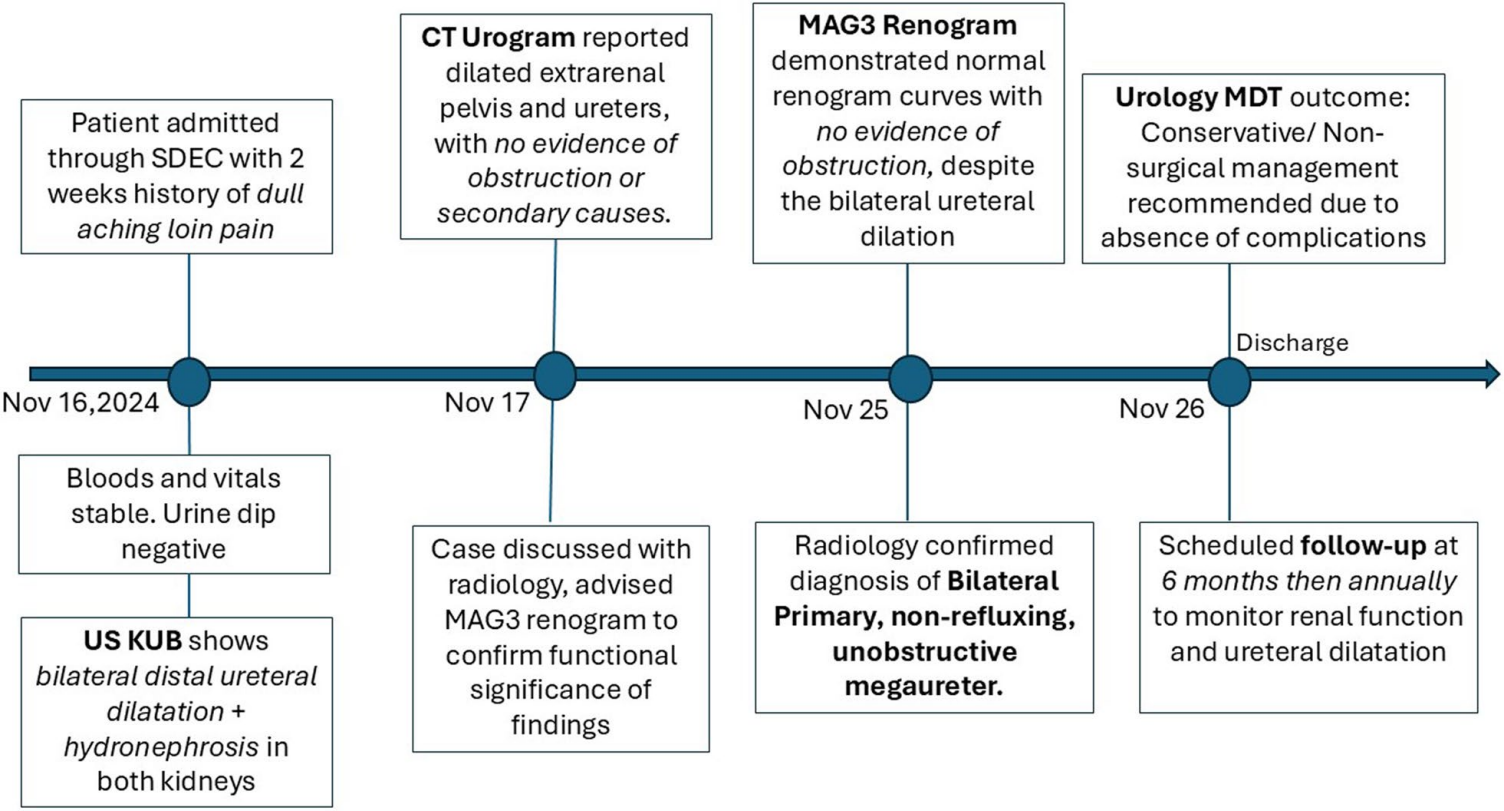
Shows a timeline figure from presentation through to planned follow-up

Given the absence of acute complications such as infection, severe obstruction, or impaired renal function, a conservative management approach was recommended. Conservative management here refers to a noninvasive approach focused on monitoring and supportive care without immediate surgical intervention. This entailed regular follow-ups with a general practitioner to monitor renal function, patient education about potential symptoms such as worsening pain, changes in urinary habits, fever, or any signs of infection that may require further evaluation, and scheduled ultrasound imaging to monitor renal function and ureteral dilation at 6 months, then annually. Surgical intervention was not deemed necessary at this time owing to the lack of significant functional impairment or progressive symptoms. (Fig. 5) The patient’s care plan emphasized the importance of vigilance for potential complications and ongoing assessment to guide future management decisions.

## Discussion

In this report, we present a rare occurrence of bilateral primary nonrefluxing unobstructed megaureter in an adult, a condition typically identified in pediatric populations and infrequently reported in adults. The uniqueness of this case lies in its presentation with vague loin pain and the absence of significant complications, such as obstruction, infection, or impaired renal function, which are commonly associated with megaureters.

This case is particularly valuable for its rarity and for demonstrating the use of noninvasive imaging modalities in reaching a diagnosis. The combination of ultrasonography, CT urogram, and MAG-3 renal scintigraphy provided comprehensive anatomical and functional evaluation, allowing for accurate diagnosis without invasive testing such as VCUG or cystoscopy. In the absence of infection, obstructive patterns, or compromised renal function, the decision to manage the patient conservatively aligns with current best practices. Highlighting this approach is educational, especially for clinicians unfamiliar with the natural history of PMU in adults. While pediatric PMU is frequently encountered and well-documented, adult presentations remain under-reported, and our case underscores the utility of structured outpatient evaluation and follow-up to avoid unnecessary interventions [6–8].

When the dilatation is not attributed to secondary causes, it is classified as primary nonrefluxing megaureter (PMU). PMU is often detected during routine antenatal ultrasounds and represents 5–10% of all cases of prenatal hydronephrosis (HN) [6, 8]. However, the incidence of prenatally diagnosed megaureter remains largely unknown, primarily owing to the challenges associated with accurate measurements using prenatal ultrasound. [4]. The classification of PMU grades is not formally standardized; however, a practical distinction can be made on the basis of the degree of dilatation: mild (7–10 mm), moderate (10–15 mm), and severe (> 15 mm). PMU is more frequently observed in males and predominantly affects the left ureter, though 25% of cases are bilateral. In unilateral cases, 10–15% are associated with an absent or dysplastic contralateral kidney [9].

The precise cause of megaureter remains unclear, although several hypotheses suggest potential abnormalities in the ureteral wall muscle fibers and irregularities in collagen composition or deposition during embryonic or fetal stages [10, 11]. In cases of nonobstructive, nonrefluxing primary megaureter, the condition may arise spontaneously or from unknown origins [12] .While there is not a definitive genetic inheritance pattern for the disease, some cases do seem to occur within families [13].

Ultrasound (US) is the primary imaging modality in the diagnostic evaluation of PMU and plays a crucial role in monitoring ureteral and renal dilation over time [6]. In addition to assessing ureteral diameter, ultrasound (US) provides valuable information about potential abnormalities in the kidney parenchyma, such as echogenicity, cystic changes, and parenchymal thickness. It also evaluates the anterior–posterior diameter of the renal pelvis, peripheral calyceal dilation, and bladder abnormalities [14].

Radiologically, hydronephrosis is either absent or present to a minimal extent in nonrefluxing, unobstructed primary megaureter [15]. While uncommon, a congenital megaureter can occur alongside congenital megacalyces, complicating the evaluation of hydronephrosis [16].

When evaluating bilateral ureteral dilatation in adults, it is crucial to consider a range of differential diagnoses beyond primary nonrefluxing unobstructed megaureter. Secondary causes must be ruled out systematically. Conditions such as neurogenic bladder, which can lead to functional urinary retention and bilateral hydronephrosis, should be considered, especially in patients with neurological symptoms or a history of spinal pathology. Obstructive causes including ureteral strictures, stones, or malignancy should also be excluded through imaging [17]. Inflammatory or infectious causes such as tuberculosis or schistosomiasis may mimic the radiographic appearance of a megaureter. In addition, acquired conditions including posterior urethral valves (rare but possible in adults), detrusor-sphincter dyssynergia, or a poorly compliant bladder due to diabetes mellitus or chronic obstruction can lead to upper tract dilation. In our patient, the absence of lower urinary tract symptoms, normal renal function, and unremarkable findings on both ultrasound and CT urogram argue against these secondary etiologies. The use of MAG-3 renography provided additional reassurance by confirming nonobstructive drainage and preserved renal function, further supporting the diagnosis of primary nonrefluxing megaureter [6, 12–14].

PMU resolution is defined as achieving a stable anteroposterior diameter of the renal pelvis measuring 10-mm or less, and/or an SFU hydronephrosis grade of 2 or less, along with ureteral dilation of less than 8 mm [18]. Although PMU often resolves spontaneously [18], it is essential to accurately establish the diagnosis by ruling out other conditions associated with megaureter, such as vesicoureteral reflux (VUR) or posterior urethral valves. Differentiating these conditions informs the clinical approach, as a wait-and-see strategy may be appropriate for VUR [19], whereas cystourethroscopy should be considered if posterior urethral valves are suspected on the basis of voiding cystourethrography (VCUG) findings [20]. Accurate interpretation of urethral cystography is crucial, as assessing even indirect signs significantly improves the diagnostic accuracy of cystography for detecting posterior urethral valves [21].

The widespread adoption of prenatal ultrasound (US) has facilitated the early identification of PMU. Many of these cases remain asymptomatic and resolve spontaneously, enabling a nonoperative management approach [4, 18, 22–25]. Resolution of PMU typically occurs early, within the first 2 years of life [1, 18], though cases have been reported resolving as late as 5 years of age and occasionally into young adulthood [26]. However, ultrasound (US) is inherently variable, with results influenced by operator interpretation and the patient’s fluid intake. Therefore, it is advisable to assess ureter diameter during physiological bladder filling, with the expected bladder capacity for age calculated using the formula: [age (years) + 1] × 30 mL [26]. In the current literature, the rate of spontaneous resolution for PMU is reported to range between 34% and 88% [27–29].

Approximately 24% of patients with PMU require surgical intervention, particularly those with a mean ureteral diameter of 17 mm or greater, while the remaining 76% experience spontaneous resolution, typically within a median timeframe of 19 months [30]. An important predictor of spontaneous resolution in PMU is baseline ureteral dilation < 11 mm, as these patients are more likely to resolve within 24 months of age. Conversely, patients with ureteral dilation > 14 mm are more likely to require surgical intervention [1]. In addition, a nonobstructive washout pattern and presentation during the prenatal or neonatal period are also significant predictors of spontaneous resolution [19]. Given the potential for long-term complications reported in the current literature [31], it is recommended to maintain long-term ultrasound follow-up, at least until puberty. The duration of follow-up should be guided by the postoperative ultrasound findings, as symptoms can emerge even after years of observation [32].

The purpose of follow-up is threefold: (i) to confirm the resolution of PMU; (ii) to detect complications or worsening conditions, thereby assessing the need for surgical intervention; and (iii) to minimize painful procedures, radiation exposure, and economic costs through accurate risk stratification [32].

This case report is limited by the absence of long-term follow-up data and the lack of a voiding cystourethrogram (VCUG) to definitively exclude vesicoureteral reflux, as the patient declined this invasive procedure. While the diagnosis of bilateral primary nonobstructing nonrefluxing megaureter was supported by imaging and renal scintigraphy, the conservative management approach also limits the ability to assess histopathological or surgical findings.

## Conclusion

In this report, we shed light on the rare occurrence of bilateral primary nonrefluxing unobstructed megaureter in the adults, demonstrating the importance of comprehensive imaging and functional assessment in diagnosis. The case emphasizes the potential for conservative management in the absence of complications and signifies the need for regular follow-up to monitor disease progression and outcomes.

## Data Availability

The data used to support the findings of this study are available from the corresponding author upon reasonable request.

## Abbreviations

CT: Computed tomography
PMU: Primary non-refluxing unobstructed megaureter
